# Cost-Effectiveness of PET/CT Surveillance Schedules to Detect Distant Recurrence of Resected Stage III Melanoma

**DOI:** 10.3390/ijerph19042331

**Published:** 2022-02-17

**Authors:** Mbathio Dieng, Robin M. Turner, Sarah J. Lord, Andrew J. Einstein, Alexander M. Menzies, Robyn P. M. Saw, Omgo E. Nieweg, John F. Thompson, Rachael L. Morton

**Affiliations:** 1 NHMRC Clinical Trials Centre, Faculty of Medicine and Health, The University of Sydney, Camperdown 2050, Australia; sally.lord@sydney.edu.au (S.J.L.); rachael.morton@sydney.edu.au (R.L.M.); 2 Biostatistics Centre, Otago University, Dunedin 9016, New Zealand; robin.turner@otago.ac.nz; 3 Seymour, Paul, and Gloria Milstein Division of Cardiology, Department of Medicine and Radiology, Columbia University Irving Medical Center and New York-Presbyterian Hospital, New York, NY 10032, USA; ae2214@cumc.columbia.edu; 4 Melanoma Institute Australia, North Sydney 2060, Australia; Alexander.M.Menzies@melanoma.org.au (A.M.M.); robyn.saw@melanoma.org.au (R.P.M.S.); Omgo.Nieweg@melanoma.org.au (O.E.N.); john.thompson@melanoma.org.au (J.F.T.); 5 Department of Medical Oncology, Royal North Shore and Mater Hospitals, North Sydney 2060, Australia; 6 Faculty of Medicine and Health, Sydney Medical School, The University of Sydney, Camperdown 2050, Australia; 7 Department of Melanoma and Surgical Oncology, Royal Prince Alfred Hospital, Camperdown 2050, Australia

**Keywords:** melanoma, follow-up, cost-benefit analysis, decision support techniques, diagnostic imaging

## Abstract

Objective: To estimate the cost-effectiveness of three surveillance imaging strategies using whole-body positron emission tomography (PET) with computed tomography (CT) (PET/CT) in a follow-up program for adults with resected stage III melanoma. Methods: An analytic decision model was constructed to estimate the costs and benefits of PET/CT surveillance imaging performed 3-monthly, 6-monthly, or 12-monthly compared with no surveillance imaging. Results: At 5 years, 3-monthly PET/CT surveillance imaging incurred a total cost of AUD 88,387 per patient, versus AUD 77,998 for 6-monthly, AUD 52,560 for 12-monthly imaging, and AUD 51,149 for no surveillance imaging. When compared with no surveillance imaging, 12-monthly PET/CT imaging was associated with a 4% increase in correctly diagnosed and treated distant disease; a 0.5% increase with 6-monthly imaging and 1% increase with 3-monthly imaging. The incremental cost-effectiveness ratio (ICER) of 12-monthly PET/CT surveillance imaging was AUD 34,362 for each additional distant recurrence correctly diagnosed and treated, compared with no surveillance imaging. For the outcome of cost per diagnostic error avoided, the no surveillance imaging strategy was the least costly and most effective. Conclusion: With the ICER for this strategy less than AUD 50,000 per unit of health benefit, the 12-monthly surveillance imaging strategy is considered good value for money.

## 1. Introduction

Melanoma patients with distant metastases in more than two organs and high tumour burden have a poor response to therapy and low survival rates [1,2]. Early treatment appears to improve the chance of survival [3,4]. Therefore, adequate and early detection of distant metastases is paramount. Frequently scheduled imaging seems an attractive tool to this end [5]. Australian and some international consensus-based guidelines recommend regular computed tomography (CT) or fludeoxyglucose positron emission tomography (FDG-PET)/CT in conjunction with clinical consultations for the follow-up of asymptomatic advanced-stage melanoma patients [6,7,8].

International guidelines for follow-up after treatment of stage III melanoma vary by country and discipline, particularly in relation to the type of imaging test recommended, the timing of imaging, and the clinical stage in which they should be performed [9]. The German guidelines recommend CT or PET/CT every 6 months for the first 3 years for resected stage III patients [10]; The United States (US) guidelines recommend imaging every 4 to 12 months for the first 5 years [8], while the United Kingdom (UK) National Institute for Health and Care Excellence (NICE) guidelines recommend surveillance imaging only if there is a clinical trial investigating the value of regular imaging, or a local policy with specific funding for imaging 6-monthly for 3 years [6]. The Australian Melanoma Guidelines (2018) state CT of the chest, abdomen, and pelvis or PET may be performed prior to definitive therapy where the detection of metastatic disease would influence management [11]. Ultrasound assessment can detect loco-regional recurrence but is not applicable for detection of systemic recurrence and is not recommended by Australian guidelines.

Furthermore, few research studies have investigated the cost-effectiveness of a surveillance imaging strategy in resected stage III melanoma patients [12,13,14]. Mostly the published studies have reported costs and benefits of one-off (baseline) imaging prior to surgery or imaging to assess treatment response in the management of distant (Stage IV) disease. As well as exploring whether annual imaging to detect systemic recurrence for resected stage III melanoma patients is more effective than no annual imaging, there is a need to investigate and establish its relative cost-effectiveness for the identification of melanoma recurrence.

The follow-up strategies in current practice were developed before potentially effective systemic therapies were available to treat advanced melanoma and also before patients with stage III melanoma received adjuvant therapy (where different adjuvant treatments have different recurrence risks, particularly in the first 1–2 years). These new therapies have significant benefits for patients and in the metastatic setting the potential to be most efficacious in patients with lower disease burden [3]. Therefore, it might be beneficial to identify recurrence earlier for patients who might benefit more from earlier systemic treatment [15,16,17]. This study aims to investigate the ‘opportunity cost’ of surveillance imaging by exploring the cost-effectiveness of four surveillance strategies for the diagnosis and treatment of distant melanoma recurrence.

Several studies have estimated the potential harms of frequent imaging, including ‘scanxiety’ [18], exposure to radiation [19], incidental findings [20], and increased financial burden [21] to patients, as well as to the healthcare system. With increasing melanoma incidence (60 cases per 100,000 people per year), the cost impact of frequent imaging for Australian healthcare is substantial [22]. According to the Australian Bureau of Statistics, the government expenditure for diagnostic imaging has doubled over the period 2000–2018 (from AUD 1159 M to AUD 3641 M per annum).

Currently, there are no high-level evidence-based follow up guidelines to assist clinicians in weighing up the costs and benefits of surveillance imaging [22]. Robust evidence of the cost-effectiveness of imaging surveillance is required for clinical practice guidelines and health policy decisions relating to post treatment surveillance.

A randomised controlled trial [23] is currently underway; however, until these results are known, a decision-analytic model drawing from the best current sources of evidence is most appropriate to inform clinical decision making [24].

This study aimed to investigate the cost-effectiveness of current surveillance imaging strategies compared with no surveillance imaging, for an accurate diagnosis of distant melanoma recurrence from a health system perspective, by modelling the relative costs and benefits over 5 years.

## 2. Methods

### 2.1. Cohort

We examined resected American Joint Cancer Committee (AJCC) stage IIIA-D [25] melanoma patients treated at the Melanoma Institute Australia (MIA) between the years 2000 and 2017 who were disease-free at the first follow-up after initial surgical treatment for melanoma. All patients were followed until the development of distant recurrence or end of follow-up schedule. The identified patients belonged to one of four follow-up categories: (1) 3–4 monthly surveillance imaging; (2) 6-monthly imaging; (3) 12-monthly imaging; or (4) no routine surveillance imaging [26].

### 2.2. Decision Model Structure

**Figure 1:**
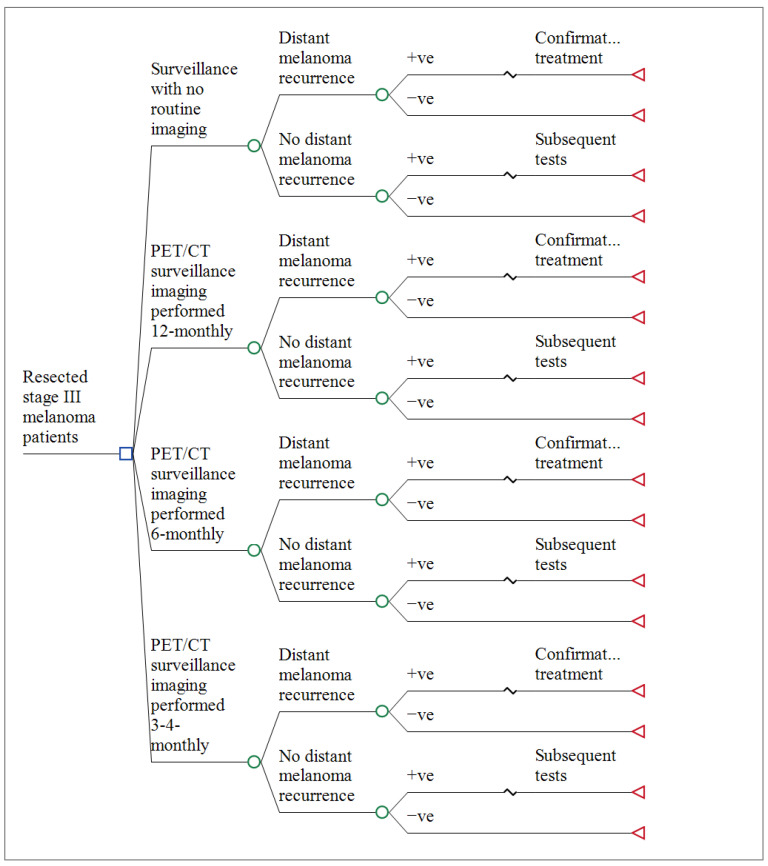
Decision model structure.

We constructed an analytic decision model to estimate the costs and benefits of follow-up with PET/CT performed 3–4 monthly, 6-monthly, or 12-monthly compared with no surveillance imaging follow up to diagnose distant melanoma recurrence. (Figure 1) The two economic outcomes evaluated were cost per distant recurrence accurately diagnosed and treated, and the cost per diagnostic error avoided. We calculated ‘distant recurrence correctly diagnosed’ as the difference between true positives and false negatives, respectively, for each branch (choice). Similarly, we calculated ‘diagnostic error avoided’ as the difference between true negatives and false positives for each branch. The model was designed to evaluate the ability of the four surveillance strategies to detect distant recurrence for each group of patients. We used an Australian health system perspective with a time horizon of 5-years and applied a 5% discount rate. Discounting is a procedure for converting costs or benefits occurring at different time points to a common measure by use of an appropriate discount rate [27]. The Australian Government recommends a discount rate of 5% is used in health technology assessment submissions [28]. The patient population was disease-free at the first follow-up visit. A detailed protocol of the economic evaluation has been published previously [26].

### 2.3. Model Assumptions

The key assumptions for the decision model were:

- Surveillance imaging was primarily used to identify distant recurrence; therefore, loco-regional recurrences were excluded.
- A first distant recurrence only occurs once. Therefore, the model simulates the costs and benefits up to the first (initial) distant recurrence.
- Based on Australian guidelines [7], patients with positive imaging test results underwent confirmatory investigations, including fine-needle aspiration biopsy (FNAB) and whole-body PET/CT (if CT only was previously undertaken).
- All patients with distant melanoma detected underwent treatment.
- The costs for unresectable stage III or stage IV melanoma treatment included diagnostic imaging, doctors’ visits, genetic testing, and pharmacotherapies where relevant, as well as costs for management of Grade 3 and 4 adverse events from treatment modalities (e.g., colitis) [29].
- Radiation-attributable malignancies from PET/CT imaging were not included because these generally take >5 years to develop.
- Costs of palliative and end of life care were excluded because patients were censored after the development of distant disease.

### 2.4. Model Inputs

The direct unit costs associated with healthcare activities in the four surveillance strategies, including PET/CT imaging, invasive procedures (e.g., FNAB and core biopsy), and clinical follow-up, were estimated from the Medical Benefits Schedule (MBS) for outpatient care and Australian-Refined Diagnosis Related Groups (AR-DRGs) for hospital admissions (Supplementary Material Table S1) [30]. The no surveillance imaging strategy costs over 5 years includes all healthcare costs, such as doctor’s visits, diagnostic tests, and procedures ordered based on symptoms or treatment.

Total costs were adjusted to 2020 Australian dollars (AUD) based on the Consumer Price Index [31]. For a whole-body PET/CT, the unit cost of PET/CT imaging was calculated as the sum of the cost of PET/low dose CT (MBS item: 61553) and adjunctive CT for the purpose of anatomic localisation (MBS item: 61505). For patients who had a positive test result (i.e., true positives and false positives), an additional cost component was added to account for the confirmatory investigations performed to verify the index imaging results. Based on the latest Australian Melanoma guidelines [11], this cost was calculated as a composite of the following investigations: fine-needle aspiration biopsy (FNAB), whole-body PET/CT, and other tests. This cost was added when the test finding was a false negative, as additional tests were often requested by the clinician to investigate the imaging findings. For the first outcome, the cost per distant recurrence correctly diagnosed and treated, patients diagnosed with stage IV disease incurred an additional cost of treatment.

### 2.5. Base Case Analysis

The base case represented a patient with melanoma stage IIIA to IIID undergoing follow-up for distant disease recurrence with surveillance imaging at the MIA. Probabilities for each branch of the decision tree were taken from the hazard rates for distant recurrence in the MIA cohort and the reported sensitivity and specificity of PET/CT [32,33]. 

The economic results were presented as the cost per distant recurrence appropriately diagnosed and treated, assuming all patients are treatable. The costs were calculated as an aggregate of follow-up surveillance costs, the cost of confirmatory tests, and the cost of treatment (applied to all patients that developed stage IV disease). For each of the outcomes, the incremental cost-effectiveness ratios (ICER) were calculated using the no surveillance imaging strategy as the comparator over a 5-year follow-up period. The ICER was compared to a willingness-to-pay (WTP) threshold of AUD 50,000 per unit of health benefit, a commonly used threshold for the value of money.

### 2.6. Sensitivity Analyses

Two major sources of uncertainty were identified for this model, uncertainty in the clinical parameters and uncertainty in the modelled calculations. Deterministic sensitivity analyses were conducted to address uncertainty for all the input clinical parameters over the range shown in Table 1. The results of one-way sensitivity analyses were illustrated using a tornado diagram [34]. To address the uncertainty in the modelled calculations, a probabilistic sensitivity analysis was conducted by assigning distributions to the model parameters to evaluate changes in the cost-effectiveness result based on the parameter distributions and their variance from the mean. Probabilities and test performance characteristics were modelled using a beta distribution, whereas costs were modelled using a gamma distribution. A Monte Carlo simulation with 10,000 replications was used for this purpose [35].

The decision model was built using TreeAge software (TreeAge Software Inc., Williamstown, MA, USA). This software package automatically generates the algorithms required to evaluate the model and choose the optimal strategy by giving weight to each possible outcome based on its probability. The cost-effectiveness analyses and the sensitivity analyses were also carried out using TreeAge, see Figure 1 for a detailed illustration of the model.

Ethics approval was not required for this cost-effectiveness study; however, ethical approval for the original cohort was obtained from the MIA research committee (MIA2016/182), the Australian Institute of Health and Welfare (EO2019-1-454), and the Royal Prince Alfred hospital X18-0144, LNR/18/RPAH/206. The economic evaluation was reported according to the CHEERS (Consolidated Health Economic Evaluation Reporting Standards) checklist [36].

## 3. Results

Table 1 presents the characteristics of patients in the three imaging regimens, and the volume of resources used. On average, each patient received 6.3 (SD3.7) CTs, PET/CT, or PET scans over 5 years and 4.8 (5.5) extra investigations conducted to confirm equivocal or false-positive findings for the 3-monthly imaging, 3.7 (2.3) scans and 4.7 (4.6) extra investigations for 6-monthly imaging, and 4.3 (2.4) scans and 3.4 (3.2) extra investigations for 12-monthly imaging. Table 2 presents the results for the outcome of cost per case of distant melanoma appropriately diagnosed and treated. For this outcome, the 12-monthly imaging schedule was both more costly and more effective than the no-imaging schedule. When compared with no routine surveillance imaging, 12-monthly imaging incurred greater incremental costs (AUD 2748) per person and resulted in an additional 4% of distant metastasis correctly diagnosed. This represented an ICER of AUD 34,362, which indicates the additional cost required to accurately diagnose and treat one person with distant melanoma. The 12-monthly imaging was less costly at AUD 52,160 per patient and more effective, with a corresponding of 92 of 100 distant metastases correctly diagnosed, than the 6-monthly imaging strategy, with a cost of AUD 77,998 per patient and a corresponding of 88 of 100 distant metastases correctly diagnosed. The 3-monthly imaging schedule was more costly and less effective than the other imaging schedules.

Table 3 presents the additional cost required to avoid a diagnosis error and shows that follow-up without imaging was less costly and more effective, with a total cost of AUD 1513 and a corresponding probability of diagnostic error avoided of 0.8832. The 3-monthly and 6-monthly PET/CT imaging strategies were more expensive options at AUD 16,268 and AUD 25,304 per patient with corresponding probabilities of diagnostic error avoided of 0.8386 and 0.7999.

### Sensitivity Analysis

**Figure 2:**
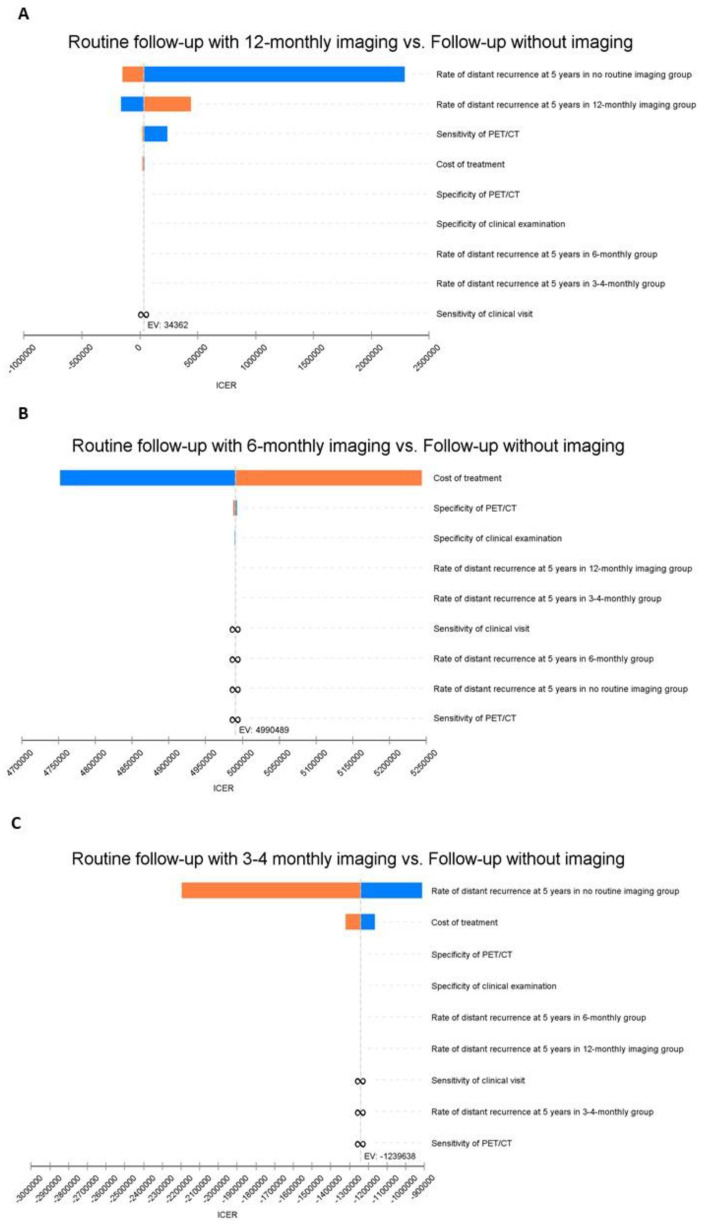
One-way sensitivity analysis (tornado) diagrams for the outcome distant disease accurately diagnosed and treated. This diagram shows the degree to which uncertainty in individual variables affects the ICER. Each bar represents the range of the individual parameter and its impact on the ICER across that range. Bars are arranged in order, with the variable with the biggest impact at the top and the variable with the smallest impact at the bottom. Other parameters also evaluated but without influence on the ICER were surveillance imaging sensitivity, specificity, and the probability of distant recurrence with the no imaging strategy. ICER: Incremental Cost-Effectiveness Ratio. (**A**) is comparing 12-monthly imaging strategy versus Follow-up without routine imaging strategy. (**B**) is comparing 6-monthly imaging strategy versus Follow-up without routine imaging strategy. (**C**) is comparing 3-4-monthly imaging strategy versus Follow-up without routine imaging strategy.

The results of one-way sensitivity analyses are displayed in a tornado diagram in which each bar represents the impact of uncertainty for the individual parameter on the ICER (Figure 2). The model was most sensitive to variations in the prevalence of distant recurrence in the no surveillance imaging and 12-monthly imaging groups and to the sensitivity of PET/CT. For example, adjusting the sensitivity of PET/CT from the base case of 79% up to 86% caused the ICER for the 12-monthly imaging strategy compared with no surveillance imaging to decrease to AUD 20,614 per case of distant melanoma accurately diagnosed and treated. Less pronounced changes in the ICER were seen when varying the costs of treatment. The cost-effectiveness results of the model were robust over a plausible range of parameter estimates.

**Figure 3:**
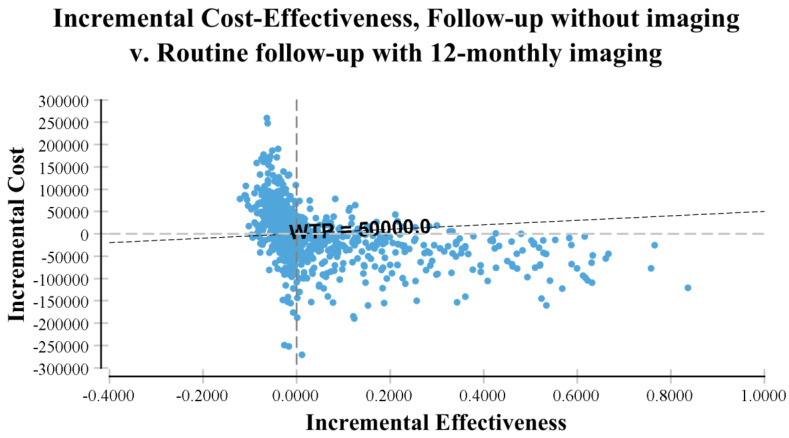
Incremental cost and effectiveness of 12-monthly PET-CT over no routine imaging strategy for the outcome of distant disease accurately diagnosed and treated. This scatter plot shows the distribution of 10,000 trials from the Monte Carlo simulation.

Overall, our probabilistic sensitivity analysis results were consistent with our base case model and provided evidence that surveillance imaging of resected stage IIIA-D melanoma patients was unlikely to be a cost-effective strategy. The probabilistic sensitivity analyses showed ICERs for imaging-based surveillance were not favourable compared with no imaging. For example, 12-monthly imaging compared with no routine imaging showed less than half (44%) of the simulations were cost-effective using a WTP threshold of AUD 50,000 per distant recurrence appropriately diagnosed and treated (Figure 3).

## 4. Discussion

Using commonly recommended surveillance imaging strategies and real-world data from a large melanoma treatment centre in Australia, our baseline model suggests 12-monthly PET/CT surveillance imaging for resected stage IIIA-D melanoma patients was slightly more expensive but more effective than a no imaging strategy with an ICER that represents good value for money. Twelve-monthly surveillance imaging was less expensive and more effective than 3 or 6-monthly surveillance imaging for the detection of distant disease. Extensive sensitivity analyses highlighted that these cost-effectiveness results were sensitive to the probability of distant recurrence at 5 years in the 12-monthly and no imaging strategies. When compared with no surveillance imaging, 12-monthly PET/CT surveillance imaging was associated with the largest increase in appropriately diagnosed distant disease versus smaller increases for 6-monthly imaging and 3–4 monthly imaging.

Our model favours less frequent imaging, which we found less expensive primarily due to fewer additional tests related to false positives. From our previous work of a well-documented stage III melanoma cohort, 42% of patients had false-positive findings and incidental findings in 21% of patients [20]. These false positives and incidental findings resulted in subsequent healthcare use, thus the elevated costs of frequent imaging.

Our findings are consistent with other studies that have analysed surveillance imaging modalities for stage II–III melanoma patients, in that frequent follow-up imaging is not cost-effective even for intermediate to high-risk patients [37,38]. However, a recent study found for AJCC stage IIC and III melanoma, 6-monthly CT scans of the chest, abdomen and pelvis were cost-effective for the early detection of metastases in the first 4 years of follow-up, over other follow-up investigations (clinical visit, brain MRI) [39]. This study stratified its population by AJCC sub-stage and compared the cost-effectiveness of CT scanning performed at baseline and first year of follow-up with imaging studies performed each subsequent year of follow-up (years 2–5). Our study population was different in that it included resected AJCC stage III patients without distant disease at their 6 or 12-month follow-up visit.

The results of the cost-effectiveness of surveillance imaging with respect to early diagnosis and treatment need to be taken together with recent research supporting survival benefits of new systemic immunotherapy [40,41]. Observational studies indicate accurate, and early detection of distant recurrence is important so that effective treatments can be commenced without delay.

Our model generated an ICER of AUD 34,362 for each additional case of accurate diagnosis and treatment of distant recurrence with a 12-monthly surveillance imaging strategy. However, an ICER of AUD 34,362 is challenging to interpret, as there is currently no established willingness to pay (WTP) threshold associated with a recurrence appropriately diagnosed and treated [42]. Based on the current arbitrary willingness to pay thresholds of AUD 50,000, this ICER suggests that 12-monthly imaging for the diagnosis of recurrent distant melanoma, in the use of every resected stage III melanoma patient, is likely to be cost-effective.

PET/CT is approved and currently being reimbursed by the Medical Services Advisory Committee (MSAC), and systemic treatment has been approved by the Pharmaceutical Benefits Advisory Committee (PBAC), meaning that the government is willing to pay for these tests and drugs. We could assume that the Australian Government would be willing to pay for the cost of a whole-body PET/CT (AUD 1128.75) for each additional accurate diagnosis, and the cost of therapeutic regimens offered to a patient diagnosed with distant metastatic disease, estimated to be AUD 115,072 [29]. Hence, this is a very wide range for the different WTP thresholds. However, there are no restrictions on the frequency of monitoring that accompanies this care.

Novel research around the standardised estimates for WTP for an appropriate diagnosis of distant recurrence is needed as immunotherapy is now given as adjuvant treatment in stage III disease to prevent distant recurrence, and that surveillance imaging is used to monitor the effectiveness of these drugs, as well as looking for the first signs of disease to treat. Future research is needed to examine the cost-effectiveness of surveillance imaging for patients with stage III melanoma undergoing adjuvant or neo-adjuvant systemic therapies.

The more frequent imaging strategies (3 or 4-monthly and 6-monthly imaging) are more costly and less effective in appropriately diagnosing distant melanoma compared with 12-monthly imaging. Indeed, the more intensive imaging strategies led to a higher number of diagnostic tests, procedures, clinic visits, and referrals to other physicians as a result of frequent false-positive findings.

The main strength of this study is that our model inputs for PET/CT test performance were taken from a recent longitudinal cohort study that assessed the performance of an imaging schedule over several years, rather than at a single (cross-sectional) time point [32]. Our study does have some limitations. First, radiation burden is important when considering the frequency of PET/CT surveillance in clinical practice. However, in this model, we did not account for radiation burden due to the limited duration of 5-years follow-up post-treatment. For lifetime models, the inclusion of lifetime attributable risk estimates for cancer incidence is important given estimates for cancer incidence following exposure to 10 mSv being highly age and gender-dependent, with young females being especially sensitive to radiation [43]. Secondly, our model did not account for survival as an effectiveness measure because the focus for surveillance imaging is on the accurate detection of disease that can lead to early treatment. The effectiveness of that treatment, and therefore, survival gains are less related to the diagnostic test but more related to the efficacy of the treatment. Furthermore, this study was specifically designed to address the impact of imaging frequency on the detection of distant recurrence and was not meant to include the survival benefit of follow-up surveillance. Indeed, the current state of the literature has limited evidence on the impact of routine surveillance imaging on survival and quality of life. In addition, analysis of this cohort showed that the initial surveillance schedule had little impact on distant disease-free survival. Randomised comparison of imaging strategies with long term follow-up is needed as in this MIA cohort, people with more advanced sub-stage were scheduled to more frequent imaging. Thirdly, the effect of PET/CT in this study was solely to assess the detection of distant recurrence in isolation to the detection of loco-regional recurrence. Lastly, concerns remain about imaging-associated patient anxiety during follow-ups for cancer patients [44]. Our model did not account for imaging-associated anxiety and psychological issues faced by patients during follow-ups. Our intent was to address policy issues for the cost-effective surveillance of resected stage III melanoma patients based on the frequency of imaging.

Our modelling suggests that 12-monthly surveillance imaging could be the most cost-effective diagnostic strategy in resected stage III melanoma patients, given current data. This work was based on a large prospective cohort study of routine PET/CT imaging in patients with stage III melanoma and is the first to quantify the costs and benefits of different imaging schedules and contribute to much-needed cost-effectiveness evidence for melanoma guidelines and health policy.

Given the changing context, there still is insufficient evidence to firmly exclude more frequent PET/CT or no routine imaging strategy as cost-effective diagnostic options for resected stage III melanoma patients. These findings must be taken together with recent research supporting that current systemic immunotherapy provides a survival benefit [10]. Indeed, this strengthens the role of early detection of metastases through intensive surveillance programmes, which eventually could lead to a significant rise in the cost associated with early diagnosis and treatment of melanoma metastases. Furthermore, it also needs to be considered that examining the value of follow-up PET/CT for detecting metastases in suspected recurrence in isolation from the same role in primary melanoma and in the adjuvant setting is not necessarily appropriate. This wider context of the use of PET/CT in follow-ups of melanoma patients needs to be considered.

Adopting the approach of less frequent imaging in resected asymptomatic patients following treatment would reduce costs and is unlikely to adversely impact the clinical outcomes of melanoma patients. Although the influence of both clinical practice guidelines and cost-effectiveness analyses on changing physician practice is variable [45,46], this study’s findings will add knowledge to the goal of reducing low-value care.

Large, prospective trials with in-built cost-effectiveness and survival studies are further needed to help guideline bodies provide efficiency-based follow-up recommendations.

## 5. Conclusions

Healthcare funding decisions are increasingly being assessed on the basis of cost-effectiveness as well as their impact on health outcomes. Among resected stage III melanoma patients, if surveillance imaging is to be performed at all, then 12 months is the most cost-effective strategy. Twelve-monthly surveillance imaging was less expensive and more effective than 3 or 6- monthly compared with no surveillance imaging, and at a WTP that is less than AUD 50,000 per patient, accurately diagnosed and treated.

## Figures and Tables

**Table 1:** Characteristic and resource use of the people included in the study by imaging schedule.

Characteristics	Imaging Schedule				
	3–4 Monthly	6-Monthly	12-Monthly	No-Imaging	Total
Total: N (%)	146 (18%)	47 (6%)	284 (35%)	346 (42%)	823 (100%)
Gender: N (%)					
Male	91 (11%)	33 (4%)	181 (22%)	230 (28%)	535 (65%)
Age at Diagnosis: Mean (SD)	59 (14.19)	54 (13.68)	52 (14.70)	61 (16.71)	57 (15.94)
Age at Diagnosis: Median (IQR)	60 (49–69)	55 (44–64)	53 (42–62)	62 (50–74)	58 (46–69)
Site of Primary: N (%)					
Head and Neck	22 (15%)	9 (19%)	43 (15%)	69 (20%)	143 (17%)
Trunk	38 (26%)	12 (26%)	102 (36%)	91 (26%)	243 (30%)
Upper Limbs	13 (9%)	4 (9%)	33 (12%)	30 (9%)	80 (10%)
Lower Limbs	28 (19%)	12 (25%)	72 (25%)	60 (17%)	172 (21%)
Occult	45 (31%)	10 (21%)	34 (12%)	95 (27%)	184 (22%)
Other				1 (0.3%)	1 (0.1%)
AJCC Stage: N (%)					
III	45 (31%)	10 (21%)	35 (12%)	101 (29%)	191 (23%)
IIIA	26 (18%)	12 (26%)	117 (41%)	42 (12%)	197 (24%)
IIIB	39 (27%)	12 (26%)	91 (32%)	74 (21%)	216 (26%)
IIIC	36 (25%)	13 (28%)	41 (14%)	129 (37%)	219 (27%)
Resource Use					
Diagnostic Imaging (surveillance)					
Count	902	172	1214		2116
Mean (SD)	6.3 (3.7)	3.7 (2.3)	4.3 (2.4)		
Range	[2–19]	[1–11]	[1–13]		
Extra investigations *					
Count	690	222	960		1872
Mean (SD)	4.8 (5.5)	4.7 (4.6)	3.4 (3.2)		
Range	[0–28]	[0–22]	[0–16]		

* No detail on sub staging.

**Table 2:** Base-case results for the cost per case of distant melanoma appropriately diagnosed and treated (2020 prices) referencing common baseline.

Strategy	Mean Cost Per Patient (AUD)	Incremental Cost (AUD)	Effectiveness *	Incremental Proportion of Distant Disease Appropriately Diagnosed and Treated *	ICER ** (AUD/Outcome)
No imaging	51,149		0.8770	-	-
12-monthly	52,160	2748	0.9181	0.0411	34,362
6-monthly	77,998	28,476	0.8824	0.0054	Dominated
3 to 4-monthly	88,387	36,860	0.8845	0.0075	Dominated ***

* Proportion of distant recurrence correctly diagnosed and treated; ** ICER: Incremental Cost-Effectiveness Ratio; *** (Over AUD 1 M).

**Table 3:** Base case results for the cost per diagnostic error avoided (2020 prices).

Strategy	Mean Total Cost Per Patient (AUD)	Incremental Cost (AUD)	Effectiveness (Diagnostic Error Avoided)	Incremental Effectiveness *
No imaging follow-up	1513		0.8832	
12-monthly imaging	9084	7571	0.8503	−0.0329
6-monthly imaging	16,268	14,755	0.8386	−0.0446
3 to 4-monthly imaging	25,304	23,791	0.7999	−0.0833

* The proportion of diagnostic error avoided. The negative sign demonstrated the no imaging strategy incurred less diagnostic error.

## Supplementary Materials

The following supporting information can be downloaded at: https://www.mdpi.com/article/10.3390/ijerph19042331/s1, Table S1: Base Case Model Parameters and Sensitivity Analysis Ranges.
