# Supplementary material for: Cost-Effectiveness of PET/CT Surveillance Schedules to Detect Distant Recurrence of Resected Stage III Melanoma
Source: Int J Environ Res Public Health. 2022 Feb 17;19(4):2331. doi: 10.3390/ijerph19042331 (PMC8872338; doi:10.3390/ijerph19042331)
Supplement: Supplementary file 1 [file ijerph-19-02331-s001.zip › ijerph-1498893-supplementary.pdf]

Supplementary Table S1: Base Case Model Parameters and Sensitivity Analysis Ranges.

Parameter	Base case value	Sensitivity analysis range	Source
**Disease characteristics**			
Rate of distant recurrence at 5 years in no routine imaging group	43%	31% - 47%	MIA cohort
Rate of distant recurrence at 5 years in 12-monthly imaging group	39%	27% - 38%	
Rate of distant recurrence at 5 years in 6- monthly imaging group	55%	29% - 59%	
Rate of distant recurrence at 5 years in 3 to 4-monthly imaging group	55%	29% - 59%	
**Diagnostic accuracy**			
Sensitivity of PET/CT	79%	70% – 86%	[33]
Specificity of PET/CT	88%	86% – 90%	[33]
Sensitivity of clinical examination	71·4%	55% – 84%	[34]
Specificity of clinical examination	99·6%	99% – 99·8%	[34]
**Costs (AUD)**			
Whole body PET/CT	1128.75	–	MBS #61553, MBS #
Ultrasound	92.75	–	MBS #55812
MRI (Brain and Head)	342.75	–	MBS #63001
X Ray (Chest and Abdomen)	80.60	–	MBS #58903, MBS #58503
FNAB	200.75	–	MBS #55054, MBS #30075
Bone Scan (whole body)	407.85	–	MBS # 61421
Clinical follow-up (Specialist)	65.20	–	MBS #116
Mutation analysis	196.35	–	MBS #73336
Core Biopsy	127.30	134.65 – 997.20	MBS #30075, AR-DRG
Serum lactate dehydrogenase (LDH)	8.25	-	MBS #66500
Cost of treatment: 12-month cost—stage III unresectable/IV			[30]
	115,072	105,208 -125,573	
